# Cryptogenic Organizing Pneumonia Secondary to Mycoplasma pneumoniae Infection: A Case Report

**DOI:** 10.7759/cureus.20623

**Published:** 2021-12-22

**Authors:** Bassem S Zeidan, Jinal K Patel, Arielle Kirk, Arnoldo Gonzalez, Imran Khan

**Affiliations:** 1 Internal Medicine, Medical Center of Trinity, Trinity, USA; 2 Infectious Diseases, Medical Center of Trinity, Trinity, USA; 3 Pulmonary and Critical Care Medicine, Medical Center of Trinity, Trinity, USA

**Keywords:** lung biopsy, video-assisted thoracoscopic surgery (vats), infectious disease pathology, pulmonary disease, cryptogenic organizing pneumonia, community aquired pneumonia, mycoplasma pneumonia, bronchiolitis obliterans organizing pneumonia, boop, cop

## Abstract

Cryptogenic organizing pneumonia (COP), previously known as bronchiolitis obliterans organizing pneumonia (BOOP), is a serious lung disease that can cause significant and long-term damage to the distal respiratory tract. Here we describe an unusual case in which an adult patient with recent mycoplasma pneumonia developed worsening lower airway disease despite initiation of multiple antibiotic therapies, illustrating the immunological effects related to *Mycoplasma* infections. It is critical that clinicians learn how to recognize early signs of COP in order to tailor appropriate therapy, reduce morbidity, and improve quality of life.

## Introduction

Cryptogenic organizing pneumonia (COP), previously known as bronchiolitis obliterans organizing pneumonia (BOOP), is a rare inflammatory lung disorder that involves the lung parenchyma and distal bronchioles, bronchiolar ducts, and alveoli [1-2]. It is clinically characterized by a preceding subacute or chronic respiratory illness and histopathologically by granulation tissue in the bronchiolar lumen, alveolar ducts, and alveoli, along with variable interstitial and airspace infiltration by mononuclear cells and foamy macrophages [1-2]. The exact pathogenesis is not known but it is often associated with systemic autoimmune disorders such as systemic lupus erythematosus (SLE) or infections like human immunodeficiency virus (HIV), cytomegalovirus (CMV), and adenovirus [1-2]. It is also associated with drugs and medications such as cephalosporins, amiodarone, cocaine inhalation, and immunosuppression following allogeneic bone marrow transplantation [1-2]. We would like to present a case of a 48-year-old male who developed COP following an acute *Mycoplasma pneumoniae* infection.

## Case presentation

A 48-year-old African American male nonsmoker with a past medical history of obesity, hypertension, and uncontrolled diabetes mellitus type 2 presented to our hospital with persistent dry cough, fever, and progressive shortness of breath over the course of two weeks. He was initially treated with oral ciprofloxacin in the outpatient setting by a telemedicine physician with no improvement. Due to a lack of improvement after four days, he presented to our emergency department for the first time. On chest radiographs, he was found to have patchy alveolar disease in the right midlung and both lung bases consistent with community-acquired pneumonia. At that time, his vitals were within normal limits and was not hypoxic, therefore he was discharged on a short course of oral azithromycin. After four additional days of failing to improve with oral antibiotics a second time, the patient returned to our hospital in which he was subsequently admitted. Upon admission, he was found to have *a Mycoplasma pneumoniae* infection, which was diagnosed via IgM titers. He was started on intravenous (IV) doxycycline and transitioned to oral doxycycline and was discharged on the remaining course of treatment. Due to further lack of improvement, the patient presented back to our facility with a low-grade fever of 100°F, mild hypoxia with oxygen saturation at 91% on room air. Lung exam revealed diffuse rales and apical rhonchi on auscultation. Laboratory findings were benign. A chest x-ray showed bilateral pulmonary opacities consistent with persisting pneumonia. He was started on intravenous antibiotics and admitted to the medical floor for further management. He remained persistently hypoxic and tachycardic despite these interventions. 

**Figure 1:**
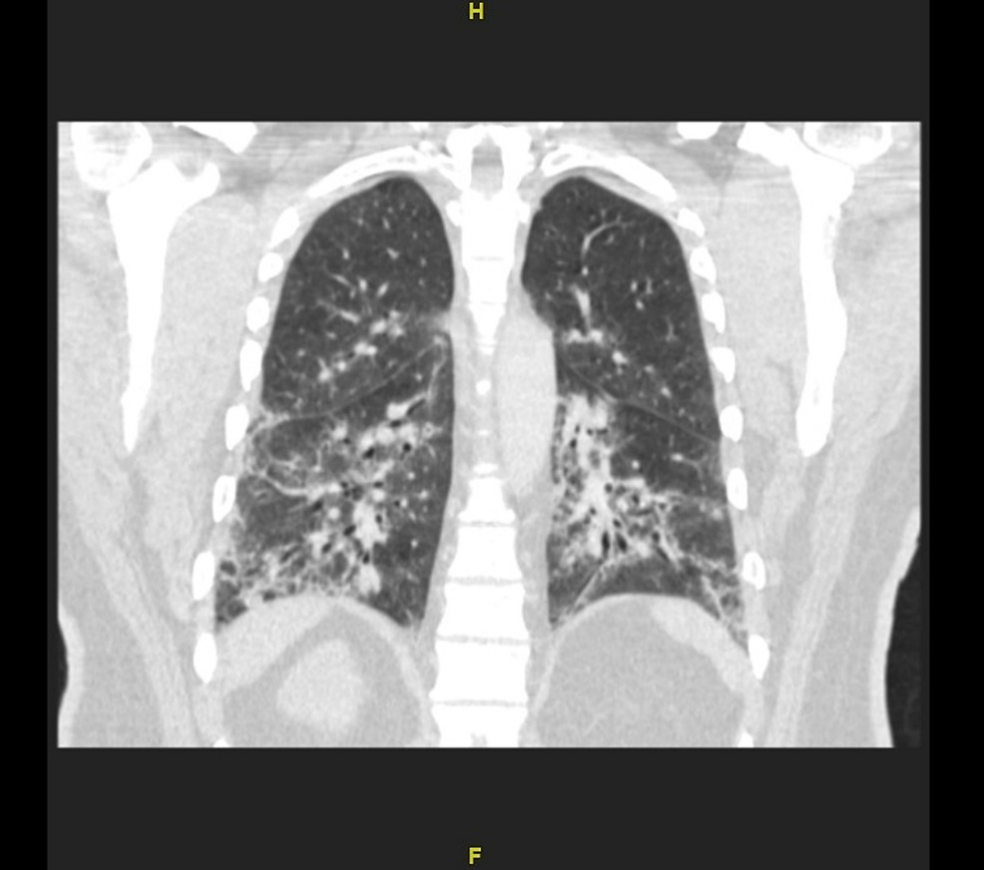
CT of the chest: Interstitial lung disease with bronchiectasis

**Figure 2:**
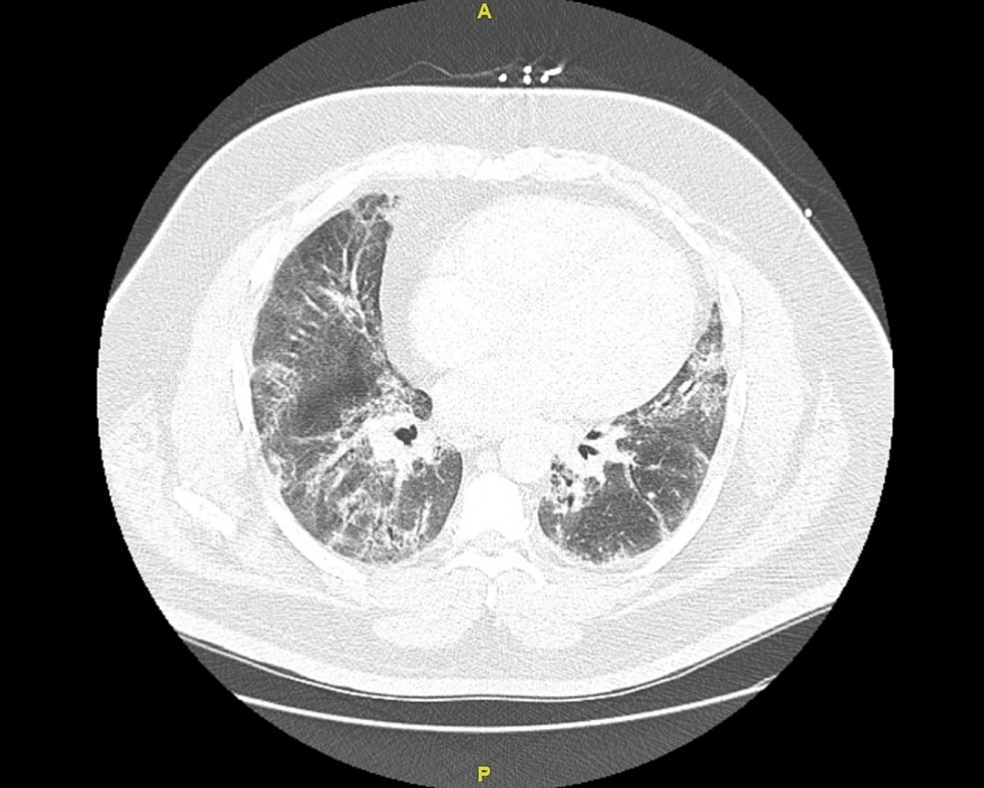
CT scan of the chest: Development of pulmonary fibrosis and bronchiectasis with scarring present at the periphery of the lungs

Over the course of his admission, his oxygen demand rose, and he required up to 9 liters of supplemental oxygen despite rigorous physical therapy, pulmonary toileting, and breathing treatments. He underwent a comprehensive workup, including repeat sputum analysis, HIV testing, and streptococcal antigen, legionella antigen, and coronavirus disease 2019 (COVID19) polymerase chain reaction (PCR) and antigen testing, all of which were unremarkable. He had gone through multiple antibiotic regimens during this admission, including doxycycline, levofloxacin, azithromycin, vancomycin, and cefepime, with minimal change. Repeat chest x-rays and a computerized tomography (CT) scan of the chest showed persistent bilateral interstitial infiltrates and bronchiectasis (Figure 1). It was evident that there was development of pulmonary fibrosis and bronchiectasis with scarring present at the periphery of the lungs (Figure 2). The patient’s oxygen needs slightly improved when he was started on high-dose steroids consisting of 60 milligrams (mg) of oral prednisone, though he remained oxygen dependent.

The medical team, including infectious disease and pulmonology, considered the possibility of a non-infectious causes for his slow improvement after starting oral steroids. They proceeded with video-assisted thoracic surgery (VATS) to obtain a lung biopsy. The biopsy report demonstrated diffuse organizing pneumonia with organizing fibrosis in the terminal airways, chronic inflammation, and increased alveolar foamy macrophages. This indicated alveolar interstitial pneumonitis, with focal bronchiolitis obliterans, also known as cryptogenic organizing pneumonia. In view of his recent diagnosis of mycoplasma pneumonia one month prior, the pathologist suggested that this may be the precipitating cause of the recent biopsy findings and the patient's current presentation. The patient and his family were informed that the recovery process would be long and would require supplemental oxygen at home and high-dose steroids. Over time his oxygen needs decreased significantly enough for him to function with a portable oxygen tank, and he was discharged home with outpatient pulmonology follow-up.

## Discussion

COP is a potentially reversible histopathologic condition and uncommon disease, but its incidence is likely higher than believed and has been rising. Contributing factors to this rise are multifactorial, including increased exposure to industrial fumes, occurrence in lung transplantation, and infections [2]. COP is caused by capillary and alveolar damage-causing leakage of plasma proteins into the alveolar lumen, fibroblast recruitment, and ultimately fibrosis. The exact mechanism is unclear, but it is theorized to be a pathogenic repair process as the body attempts to repair damaged lung tissue [3]. It is associated with systemic autoimmune disorders, toxins, and, rarely, infections like that of *Mycoplasma pneumoniae*. 

*Mycoplasma pneumonia* infection is seldom associated with post-infectious COP, and reports of severe mycoplasma pneumonia in the adult population are sparse. [4] In the literature review, we were able to find 10 adult inpatients identified with a diagnosis of bronchiolitis in which three had a definitive clinical diagnosis of *Mycoplasma*-associated bronchiolitis. [5] Nearly all of these patients presented with progressive dyspnea and cough and tested positive for *Mycoplasma pneumoniae* through either IgM titers or PCR. Imaging findings of COP are less consistent. Typical findings include patchy ground-glass opacities in a subpleural and/or peribronchovascular distribution (80% of cases), bilateral basal airspace consolidation (71% of cases), and bronchial wall thickening and cylindrical bronchial dilatation in areas of air bronchogram (71% of cases) [1]. In our patient, bilateral interstitial infiltrates and bronchiectasis were present on the CT scan of the chest. In reported cases of *Mycoplasma*-related COP, patients received antibiotics but experienced the most improvement following initiation of steroids [5]. Patients had histories, laboratory and histopathological findings on biopsy, and therapeutic responses to steroids similar to this case. 

Patients with *Mycoplasma*-induced organizing pneumonia responded well to long-term steroid therapy (for six to 24 months) [6]. COP is exquisitely sensitive to oral corticosteroids but if the diagnosis is not considered in such patients and appropriate treatment is not instituted early, COP often leads to prolonged hospital admission with considerable morbidity [7]. The classic paper by Epler describing COP/BOOP concluded 1/3 improved, 1/3 worsened and 1/3 stayed the same [8]. Nevertheless, COP can be effectively treated a second and third time with the previously effective dosage level of prednisone [9].

## Conclusions

Our case demonstrates the importance of early recognition of COP as a potential diagnosis in patients with suspected pneumonia that have failed to improve with antibiotic therapy. Early recognition of COP/BOOP will prompt more aggressive investigation to confirm diagnosis and tailor steroid therapy early on in the disease process, which will ultimately lead to reduced morbidity, hospital stay, and improved quality of life.
